# Improving the efficiency of drug resistant tuberculosis treatment trials: a time-to-event alternative marker for bacteriological response and adaptive minimization for randomization

**DOI:** 10.1186/s12874-025-02697-w

**Published:** 2025-11-25

**Authors:** Elise De Vos, Annelies Van Rie, Steven Abrams

**Affiliations:** 1 Department of Family Medicine and Population Health, University of Antwerp, Antwerp, Belgium; 2 Department of Clinical Sciences, Institute of Tropical Medicine, Antwerp, Belgium; 3 Data Science Institute, Interuniversity Institute for Biostatistics and statistical Bioinformatics, Hasselt University, Diepenbeek, Belgium

**Keywords:** Sample size, Tuberculosis, Multidrug-Resistant, Clinical trial, phase III, Epidemiologic methods, Random allocation, epidemiologic research design

## Abstract

**Background:**

Establishing the efficacy of new treatments for rifampicin-resistant tuberculosis (RR-TB) is challenging due to the long-term clinical endpoints of two-year relapse-free survival. This study aimed to evaluate the effect of an alternative indicator of treatment response on sample size requirements and the use of a minimization strategy for randomization.

**Methods:**

Sample size estimates were compared when based on the commonly used endpoint of the proportion of patients achieving stable culture conversion (SCC) at 12 weeks versus a novel but corresponding indicator of treatment response based on a model of changes in mycobacterial load (MBL) over time. The non-linear mixed effects model, calibrated using data from a RR-TB cohort in the same setting, included a longitudinal MBL decline, a probabilistic component for mycobacteria presence in sputum, and a time-to-event model for culture positivity. Data were simulated for a prespecified treatment effect to compare the power of detecting the treatment effect for various sample sizes when using the commonly used endpoint and alternative indicator of treatment response. Additionally, the impact of random patient allocation versus a minimization strategy for randomization on covariate imbalance was assessed.

**Results:**

To achieve 80% power, 410 individuals were needed using the commonly used endpoint versus 110 participants when using the non-linear mixed effects model, corresponding to a 73% reduction in sample size. A small sample size results in high baseline covariate imbalance with random treatment group allocation, with a median relative imbalance of 0.104 for 110 participants versus 0.053 for 410 participants. This imbalance was reduced to 0.036 for 110 participants when an adaptive minimization procedure was implemented.

**Conclusion:**

Using a model of mycobacterial burden changes over time as an alternative indicator of treatment response, combined with a minimization procedure during the randomization process, significantly reduced the sample size which could, if validated, enhance the efficiency of RR-TB clinical trial design.

**Supplementary Information:**

The online version contains supplementary material available at 10.1186/s12874-025-02697-w.

## Introduction

 Drug-resistant tuberculosis remains an important global health problem. Treatment of multi-drug resistant tuberculosis (MDR-TB) and rifampicin-resistant RR-TB is challenging with an estimated success rate of 68% globally in 2023 [1]. It is also accompanied by frequent occurrence of adverse events, low treatment compliance, and high costs. Rapid identification of resistance and swift initiation of a drug regimen containing at least four effective drugs is quintessential for an optimal treatment outcome [2].

Until 2018, RR-TB treatment guidelines were based on expert opinion and observational data [3]. In 2016, the STREAM trial was the first phase III randomized clinical trial (RCT) evaluating a novel treatment regimen for MDR-TB [4]. Since then, several large RCTs have been completed or are ongoing to investigate the efficacy of new and/or repurposed drugs, optimal drug combinations, drug dosing, and treatment duration. Examples are the endTB and endTB-Q trials (NCT04062201), BEAT Tuberculosis (NCT04062201), SimpliciTB (NCT03338621), NEXT trial (NCT02454205), and TB PRACTECAL (NCT02589782).

Establishing the efficacy of a novel drug, drug regimen or treatment duration poses significant challenges as the preferred clinical endpoint in phase 3 trials is two-year relapse-free survival. Alternative endpoints commonly used in TB trials include the proportion of culture negative survival at month 6 (InDEX trial, NCT03237182), the proportion of patients reaching sputum culture conversion at a specified timepoint (e.g., week 8 to determine which regimen to drop from the phase 3 TB PRACTECAL trial), or culture status at a predetermined timepoint as part of a composite measure for the clinical outcome (e.g., week 73 after randomization in endTB-Q trial or at the end of treatment in the BEAT Tuberculosis trial). Unlike for other diseases such as HIV (viral load) or cancer (tumor shrinkage), none of the alternative end points for drug resistant TB have been validated as surrogate markers according to Prentice’s criteria: namely, by demonstrating that they reliably predict long-term treatment success and that a test of no treatment effect on the surrogate implies no treatment effect on the true clinical endpoint across different populations and regimens [5].

Recently, a novel semi-mechanistic non-linear mixed effects model was used to compare changes over time in mycobacterial load (MBL) between two treatment regimens [6]. The model developed by Chigutsa et al. [7] and extended by Svensson et al. [6] includes a latent longitudinal representation of the evolution of MBL in patients, the probability of bacterial presence in a sputum sample, and a time-to-event model describing time to culture positivity in liquid culture in relation to time since treatment initiation. In contrast to the commonly used endpoint (i.e., proportion of patients with stable culture conversion at a specific time point after diagnosis or treatment initiation) which only comprises a single datapoint per patient based on serial culture data, the model uses all culture data collected at baseline and during treatment to quantify the treatment effect.

In this work, we compare simulated model-based change in MBL over time to 12-week culture conversion, with the aim to evaluate the potential of this approach to improve the efficiency of phase 3 trial design. We hypothesized that the gain in information by exploiting the longitudinal nature of the culture data could reduce the sample size required to identify a significant treatment effect. We explore the potential utility of change in MBL as an early marker of treatment response, using a hypothetical framework to assess how MBL-based endpoints might impact sample size requirements. While concept of using longitudinal microbiological data to increase statistical power in TB trials is not new [8–10], our work expands on this by considering the implications of MBL as an alternative indicator of treatment response. As such, we aim to contribute to the broader discussion of optimizing trial design for TB treatment evaluation, while awaiting validation of MBL as a surrogate marker for relapse-free survival.

To avoid bias and to ensure that treatment assignment is not based on patients’ prognostic factors; RCTs randomize participants to a treatment or reference group (standard treatment or placebo). Simple randomization can lead to unbalanced groups, especially for RCTs with small sample sizes [11]. A minimization strategy for randomizing treatment allocation has been shown to be an effective way to minimize the imbalance in baseline covariates, inevitably created by simple randomization procedures; but has not yet been widely implemented in RR-TB trial designs [12–14].

In this manuscript, we use a hypothetical framework to evaluate the effect of (1) an alternative marker of treatment response and (2) use of minimization instead of standard randomization procedure on the design of an RCT of whole-genome sequencing-guided individualized RR-TB treatment. Specifically, we provide proof-of-concept for the use of the change in mycobacterial load estimated by the semi-mechanistic non-linear mixed effects model as an alternative marker of treatment response on the sample size required. Next, we compare a minimization strategy for treatment allocation with standard randomization on the imbalance in important covariates in an RCT with a small sample size.

## Methods

### The SMARTT trial

The Sequencing Mycobacteria and Algorithm-determined Resistant Tuberculosis Treatment trial (SMARTT-trial, NCT05017324) is a pragmatic single-blinded randomized controlled medical device trial evaluating the efficacy of an automated Whole Genome Sequencing (WGS)-guided individualized treatment recommendation for RR-TB [15]. Adults diagnosed with pulmonary RR-TB in the Free State province in South Africa are randomized at a 1:1 ratio to either the experimental (WGS) arm or the control arm in which patients are treated according to the local standard of care (SOC). All participants start a standard RR-TB treatment regimen upon RR-TB diagnosis. Treatment is individualized based on a sequencing-based treatment recommendation (experimental arm) [16] or based on results of line probe assays and phenotypic drug susceptibility tests (control arm). Treatment response is monitored using sputum cultures at weeks 2, 3, 4, 5, 6, and 8, and monthly thereafter until treatment completion.

### Non-linear mixed effects model

The model inspired by the work of Svensson et al. consists of three components [6, 7]: [1] a model for the mycobacterial load (MBL) as a function of time since the start of treatment ($\:{\text{T}}_{\text{S}\text{T}})$, where MBL is derived from serial sputum samples analysed in liquid culture using the time-to-positivity output from the mycobacterial growth indicator tube (MGIT; Becton Dickinson, Sparks, MD, USA) system; [2] a probabilistic model for the presence of mycobacteria (defined by MGIT culture positivity) in relation to the time on treatment, and [3] a model for the time to culture positivity as a function of the time since inoculation in the MGIT system $\:\left({\text{T}}_{\text{G}}\right)$ [6].

A mono-exponential model for the MBL decline in sputum specimens on treatment is expressed as

$$\:\text{M}\text{B}\text{L}\left({\text{t}}_{\text{i},\text{S}\text{T}}\right)=\:{\text{A}}_{\text{i}}\text{e}\text{x}\text{p}\left(-{\upalpha\:}{\text{t}}_{\text{i},\text{S}\text{T}}\right),$$

where $\:{\text{t}}_{\text{i},\:\text{S}\text{T}}$ is the time since the start of treatment for individual $\:i,\:{\upalpha\:}\:$represents the rate of killing of mycobacteria through treatment, and $\:{\text{A}}_{\text{i}}$ denotes the baseline number of mycobacteria present in the sputum specimen of individual$\:\:i$ = 1, …, n (with n representing the sample size). $\:{\text{A}}_{\text{i}}$ is expressed through a non-linear function:

$$\:{\text{A}}_{\text{i}}=\text{M}\text{B}{\text{L}}_{0}{\left(\frac{\text{T}\text{T}{\text{P}}_{0,\text{i}}}{\text{T}\text{T}{\text{P}}_{0,\text{p}\:}}\right)}^{{\upgamma\:}}$$

where $\:\text{M}\text{B}{\text{L}}_{0}$ is the estimated number of mycobacteria present in the inoculum at the start of the treatment, $\:\text{T}\text{T}{\text{P}}_{0,\:\text{i}}\:$is the individual’s observed time to positivity (TTP) at baseline, and $\:\text{T}\text{T}{\text{P}}_{0,\:\text{p}}\:$represents the population median TTP at baseline. The model parameter $\:{\upgamma\:}$ determines the extent of individual variation in MBL at baseline relative to the median population value of the baseline MBL. Inter-individual variation in the decline of MBL is achieved by replacing the constant exponential MBL decay rate $\:{\upalpha\:}$ by an individual-specific rate:

$$\:{{\upalpha\:}}_{\text{i}}=\frac{\text{ln}\left(2\right)}{\text{H}{\text{L}}_{\text{i}}},$$

where HL_i_ is modeled in terms of the patient-specific half-life of MBL as

$$\begin{aligned}{\mathrm{HL}}_{\mathrm i}\;&=\;\mathrm{mHL}\left(1\;+\;{\upbeta}_{\mathrm{SOC}}\right) \\ & \quad \;\; \left(1\;+\;{\upbeta}_{\mathrm{WGS}}{\mathrm{WGS}}_{\mathrm i}\right)\;\exp\left({\uppsi}_{\mathrm i}\right)\,. \end{aligned}$$

In this equation, $\:\text{m}\text{H}\text{L}$ is the mean half-life, $\:{{\upbeta\:}}_{\text{S}\text{O}\text{C}}$ is the effect on MBL half-life induced by SOC RR-TB management$\:,\:{{\upbeta\:}}_{\text{W}\text{G}\text{S}}$ represents the additional effect on the half-life induced by the trial intervention, and $\:{\text{W}\text{G}\text{S}}_{\text{i}}$ is a dummy variable indicating whether patients receive SOC ($\:{\text{W}\text{G}\text{S}}_{\text{i}}=0$) or WGS-guided individualized treatment ($\:{\text{W}\text{G}\text{S}}_{\text{i}}=1$). Because patients randomized to the WGS arm initiate SOC treatment while awaiting WGS results, the decay in MBL since diagnosis also accounts for the time that patients (in both arms) received a SOC treatment regimen. Individual heterogeneity in MBL half-life is induced by normally distributed random effects $\:{{\upeta\:}}_{\text{i}}\:\sim\text{N}\left(0,{{\upsigma\:}}_{\text{i}}^{2}\right)$ which relate to $\:{{\uppsi\:}}_{\text{i}}$ as follows:

$$\:{{\uppsi\:}}_{\text{i}}=\frac{{\text{exp}\left({{\upeta\:}}_{\text{i}}\right)}^{\text{B}\text{X}\text{P}}-1}{\text{B}\text{X}\text{P}},\:$$

with BXP representing a Box-Cox transformation parameter.

The second component of the model is the time-specific probability of mycobacterial presence, denoted by $\:{\text{P}}_{\text{i},\:\text{p}\text{o}\text{s}}\left({\text{t}}_{\text{i},\:\text{S}\text{T}}\right)$, in the sputum specimen of individual $\:i$, collected at time $\:{\text{t}}_{\text{i},\:\text{S}\text{T}}$, given the MBL of individual $\:i$ at that time point. This can be described using an $\:{\text{E}}_{\text{m}\text{a}\text{x}}$ model including the maximal risk of bacterial presence, i.e., $\:{\text{P}}_{\text{m}\text{a}\text{x}}$, and the MBL value for which $\:{\text{P}}_{\text{i},\:\text{p}\text{o}\text{s}}\left({\text{t}}_{\text{i},\:\text{S}\text{T}}\right)$ corresponds to 50% of $\:{\text{P}}_{\text{m}\text{a}\text{x}}$ (i.e., a quantity hereunder referred to as $\:\text{M}\text{B}{\text{L}}_{50}$):

$$\:{\text{P}}_{\text{i},\:\text{p}\text{o}\text{s}}\left({\text{t}}_{\text{i},\:\text{S}\text{T}}\right)=\:\frac{{\text{P}}_{\text{m}\text{a}\text{x}}\:\text{M}\text{B}\text{L}\left({\text{t}}_{\text{i},\:\text{S}\text{T}}\right)\:{\text{w}}_{\text{i}\text{j}}}{\text{M}\text{B}\text{L}\left({\text{t}}_{\text{i},\:\text{S}\text{T}}\right)\:{\text{w}}_{\text{i}\text{j}}+\text{M}\text{B}{\text{L}}_{50}}\:,$$

where $\:{\text{w}}_{\text{i}\text{j}}=\text{e}\text{x}\text{p}\left({\text{z}}_{\text{i}\text{j}}\right)$ represents the inter-occasion variability in the sputum sampling procedure (only estimable in case of repeated sampling) and $\:{\text{z}}_{\text{i}\text{j}}\:\sim\:\text{N}(0,{{\upsigma\:}}_{\text{S}}^{2})$.

The third component of the model describes mycobacterial growth in the MGIT system. The number of bacteria present at the time after inoculation $\:{\text{t}}_{\text{G}}$, denoted by $\:\text{B}\left({\text{t}}_{\text{G}}\right)$, is described using a logistic growth model with inoculum size corresponding to the MBL:

$$\:\frac{\text{d}\text{B}\left({\text{t}}_{\text{G}}\right)}{\text{d}{\text{t}}_{\text{G}}}\:=\:\text{B}\left({\text{t}}_{\text{G}}\right){\text{k}}_{\text{G}}[{\text{B}}_{\text{m}\text{a}\text{x}}\:-\:\text{B}({\text{t}}_{\text{G}}\left)\right],$$

with initial condition $\:\text{B}\left(0\right)\:=\:\text{M}\text{B}\text{L}\left({\text{t}}_{\text{i},\:\text{S}\text{T}}\right)$, and where $\:{\text{k}}_{\text{G}}$ is the growth rate and $\:{\text{B}}_{\text{m}\text{a}\text{x}}$ is the maximal mycobacteria-carrying capacity of the system. The logistic equation is solved analytically to have the following solution:

$$\:\text{B}\left({\text{t}}_{\text{G}}\right)=\frac{{\text{B}}_{\text{m}\text{a}\text{x}}\:}{1\:+\:\text{C}\:\text{e}\text{x}\text{p}(-{\text{k}}_{\text{G}}\:{\text{B}}_{\text{m}\text{a}\text{x}}\:{\text{t}}_{\text{G}})},$$

where

$$\:\text{C}\:=\:\frac{{\text{B}}_{\text{m}\text{a}\text{x}}\:-\:\text{B}\left(0\right)}{\text{B}\left(0\right)}\:.$$

The hazard function is assumed to be directly proportional to the number of mycobacteria present in the MGIT tube, i.e., $\:{\uplambda\:}\left({\text{t}}_{\text{G}}\right)\equiv\:\:{\text{h}}_{\text{s}\text{c}\text{a}\text{l}\text{e}}\:\text{B}\left({\text{t}}_{\text{G}}\right)$, with $\:{\text{h}}_{\text{s}\text{c}\text{a}\text{l}\text{e}}$ estimated from the observed data.

In conclusion, we model the *MBL* at time $\:{\text{t}}_{\text{i},\text{S}\text{T}}$​ (days since treatment start) as follows:

$$\begin{aligned} &\:\text{MBL}\left({\text{t}}_{\text{i},\text{ST}}\right)=\:\text{MBL}_{0}{\left(\frac{\text{TTP}_{0,\text{i}}}{\text{TTP}_{0,\text{p}\:}}\right)}^{\upgamma}\\ & \exp\left(-\frac{\ln\left(2\right)}{\text{mHL}\left(1+{{\upbeta}}_{\text{SOC}}\right)\left(1+{{\upbeta}}_{\text{WGS}}{\text{WGS}}_{\text{i}}\right)\exp\left(\frac{{\exp\left({{\upeta}}_{\text{i}}\right)}^{\text{BXP}}-1}{\text{BXP}}\right)}{\text{t}}_{\text{i},\text{ST}}\right),\end{aligned}$$

where $\:{\text{W}\text{G}\text{S}}_{\text{i}}$ is an indicator for whole-genome sequencing (experimental arm), $\:{{\upbeta\:}}_{\text{S}\text{O}\text{C}}$​ and $\:{{\upbeta\:}}_{\text{W}\text{G}\text{S}}$​ represent the treatment effect on the MBL half-life,$\:\text{m}\text{H}\text{L}$ is the reference half-life, $\:{{\upeta\:}}_{\text{i}}\:$captures individual-level random variability (exponential Box-Cox transformation applied as detailed in the main text), and $\:\text{T}\text{T}{\text{P}}_{0,\text{i}}\:$and $\:\text{T}\text{T}{\text{P}}_{0,\text{p}\:}$​ represent the initial TTP of patient i and a population reference, respectively. For identifiability reasons, $\:{{\upbeta\:}}_{\text{S}\text{O}\text{C}}$ is constrained to be equal to 0.5 in order to be able to estimate $\:{{\upbeta\:}}_{\text{W}\text{G}\text{S}}$. A choice for $\:{{\upbeta\:}}_{\text{S}\text{O}\text{C}}$ changes the interpretation of mHL (e.g., $\:{{\upbeta\:}}_{\text{S}\text{O}\text{C}}$ = 0 implies that mHL corresponds to the half-life in the SOC arm) and does not affect the interpretation of the parameter $\:{{\upbeta\:}}_{\text{W}\text{G}\text{S}}$ representing the treatment effect. In case, $\:{{\upbeta\:}}_{\text{W}\text{G}\text{S}}=0$ the WGS treatment does not lead to a different half-life. Thus, in summary, the treatment assignment affects the MBL decline through its multiplicative impact on the half-life, which in turn determines the rate of decline of mycobacterial burden over time.

Estimating the different model parameters from the mechanistic model will be done using maximum likelihood inference for the marginal likelihood function related to the repeated time to positivity measurements for patients in both treatment arms. The reader is referred to the Supplementary Material for more details regarding the estimation procedure. In the next section, we will elaborate about the different steps related to the sample size calculation in which the estimation procedure is key to determine the achieved power, given a sample size under consideration.

### Sample size calculation: overview of methods

**Fig. 1:**
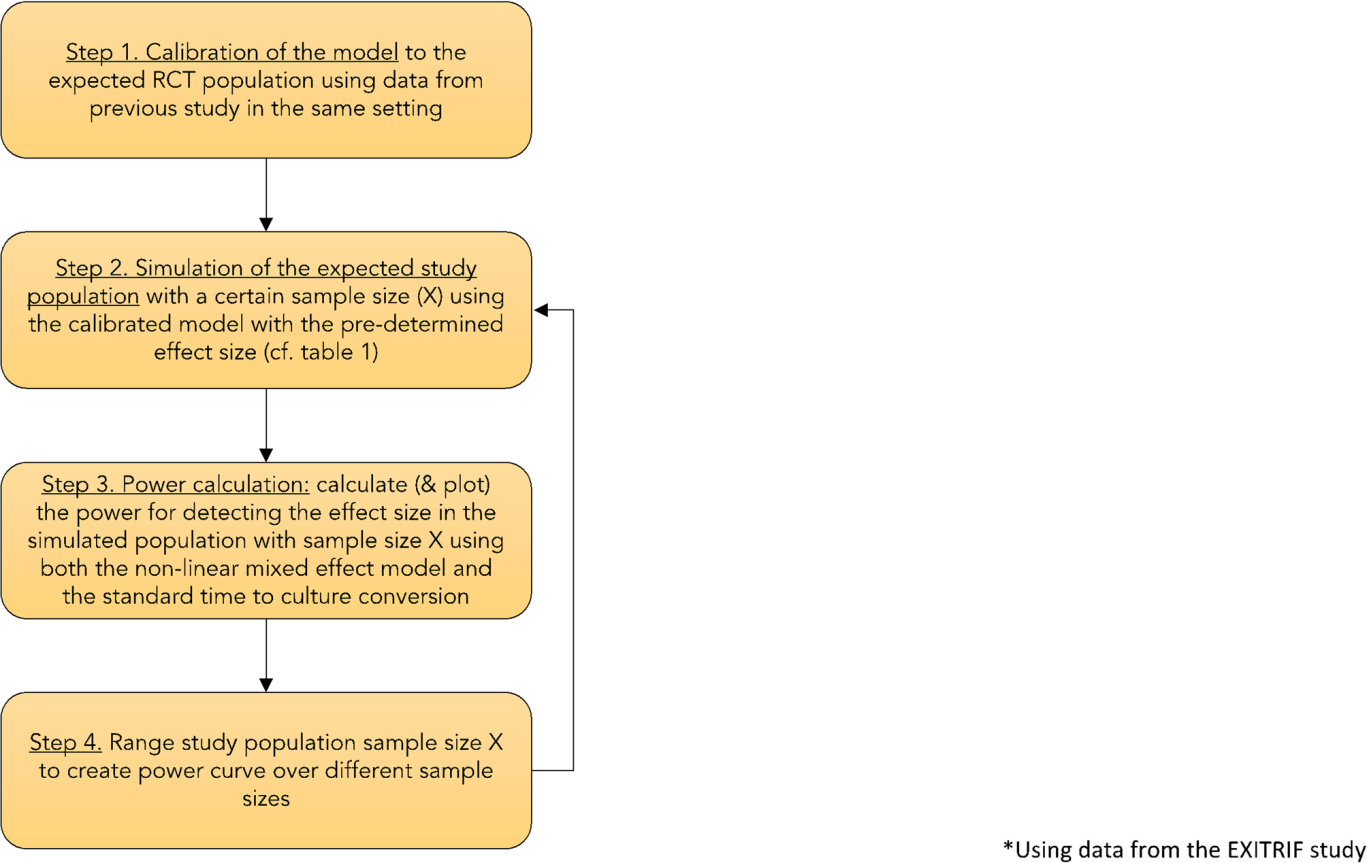
Overview of the simulation procedure used to determine the optimal sample size for the SMARTT trial

The sample size estimation for the SMARTT trial, using the non-linear mixed effects model as an alternative marker of treatment response, consisted of several steps (Fig. 1). In Step 1 we re-calibrated the model developed by Svensson et al. to the population expected to be recruited in the SMARTT trial. In Step 2, using the recalibrated model parameters and the fixed assumed treatment effect size of the intervention, we simulated the trial population for a pre-determined sample size X. In Step 3, we calculated the power to detect the assumed effect size in the simulated trial population with sample size X, using both the alternative marker of treatment response (based on the non-linear mixed effect model for changes in mycobacterial burden over time) and the commonly used endpoint (i.e., proportion of patients reaching stable culture conversion at 12 weeks). In Step 4, we repeated Step 2 to 3 for a range of sample size values to create a power curve describing the relation between the sample size and achieved power.

#### Step 1: calibration of the model

The study population used by Svensson et al. [6] consisted of patients with a first episode of pulmonary TB (0% history of TB treatment), of which 30% were HIV positive and 71% were female [6]. Characteristics of participants enrolled in the SMARTT trial are expected to differ; we expect 50% of participants to have a history of TB treatment, 78% to be HIV co-infected and 50% female, as observed in a prospective observational RR-TB cohort in South Africa (EXIT-RIF study) [17, 18]. We therefore re-calibrated the model by Svensson et al. using data from 496 EXIT-RIF study participants. Based on the distribution of baseline time to MGIT culture positivity (TTP) observed in the EXIT-RIF study, we re-estimated the population mean mycobacterial load at baseline ($\:\text{M}\text{B}{\text{L}}_{0}$), the parameter $\:{\upgamma\:}$ governing individual variation in baseline MBL, and the scaling factor $\:{\text{h}}_{\text{s}\text{c}\text{a}\text{l}\text{e}}$. We did not re-estimate the growth rate $\:{\text{k}}_{\text{G}}$, as we assume no meaningful differences in distributions of M.tb strain lineages and sublineages between the EXIT-RIF and SMARTT study populations, as both studies were conducted in the same province of South Africa over similar time periods. Values for $\:{\text{B}}_{\text{m}\text{a}\text{x}}$ (maximal bacteria-carrying capacity of the system) and $\:\text{M}\text{B}{\text{L}}_{0}$ were assumed to be proportional to the reported values in [6], with $\:\text{M}\text{B}{\text{L}}_{0}^{\text{*}}=\text{f}\:\times\:\:{\text{M}\text{B}\text{L}}_{0}$ and $\:{\text{B}}_{\text{m}\text{a}\text{x}}^{\text{*}}\:=\:\text{f}\:\times\:\:{\text{B}}_{\text{m}\text{a}\text{x}}$ representing the respective quantities for the SMARTT study population.

#### Step 2: simulation of the expected study population

To simulate the effect of WGS-guided individualized RR-TB treatment compared to SOC on the mean half-life of MBL, we first generated random individual-specific MBL values with time since diagnosis. We differentiated between time since diagnosis and time since individualized treatment because all participants first initiate a standard RR-TB treatment regimen and treatment is only individualized upon availability of WGS results. According to the study protocol, WGS results were expected to be available within 2 weeks after diagnosis (DNA extraction directly from sputum) in 50% of participants and between 2 and 4 weeks after diagnosis (DNA extraction from primary liquid cultures) for the remaining patients. For the latter, the time of the start of individualized treatment was selected randomly from a uniform distribution on the interval ranging from 15 to 28 days after diagnosis.

According to the study protocol, participants have sputum sampling performed at baseline, week 2, 3, 4, 5, 6, and 8 and monthly thereafter until treatment completion. As this is a pragmatic trial embedded in a routine public health setting, we expect that 50% of participants will have one or more missing sputum samples. We therefore randomly generate a compliance indicator with a probability of no sputum sample collected at week 3 and 5 equal to 50%.

The number of bacteria $\:\text{B}\left({\text{t}}_{\text{G}}\right)$ in the MGIT tube is calculated by generating a probability of mycobacterial presence in sputum samples at a given sampling time (since diagnosis) and given bacterial presence [6]. As the hazard of testing culture positive is assumed to be directly proportional to the number of bacteria in the MGIT tube, we simulated event times by solving the following equation:

$$\begin{aligned} \mathrm{u}\;&=\;\mathrm{S}\left(\mathrm{t}_{\mathrm{G}}\right)\;=\;\exp\left(-\int_{0}^{\mathrm{t}_{\mathrm{G}}} \lambda\left(\mathrm{t}\right)\mathrm{dt}\right)\;\\ &=\;\exp\left(-\int_{0}^{{\mathrm{t}}_{\mathrm{G}}}{\mathrm{h}}_{\text{scale}}\mathrm{B}\left(\mathrm{t}\right)\mathrm{dt}\right). \end{aligned}$$

We randomly generated values $\:\text{u}$ from a uniform distribution on the unit interval to solve the aforementioned equation for $\:{\text{t}}_{\text{G}}$ (giving rise to event times between 0 and 50 days). Event times larger than 50 days are right-censored in the data generating process; as these observations are by definition culture negative in the MGIT system.

Based on the generated TTP after inoculation at different time points, stable sputum culture conversion could be estimated and used to calculate the commonly used endpoint of the proportion of patients reaching stable sputum culture conversion at 12 weeks.

#### Step 3: power calculation

We then fitted the semi-mechanistic model to each simulated dataset to perform the power calculations. Specifically, for each simulated trial with a given sample size, a one-sided Wald test was applied to test the null hypothesis of no superior treatment effect of individualized treatment ($\:{{\upbeta\:}}_{\text{W}\text{G}\text{S}}$ = 0) compared to SOC. Power was calculated as the proportion of simulated trials meeting the criterion of sufficient evidence; defined as a one-sided p-value below the nominal significance level of 0.05. Additional simulation results indicated that the Type I error rate ranged from 7.6% to 8.8% across sample sizes; which corresponds to empirical confidence levels between 91.2% and 92.4%, only slightly below the nominal 95% level. The simulation procedure was repeated M = 250 times for each sample size. Further details on model fitting are provided in the Supplementary Materials.

We also computed the achieved power when using the proportion of participants reaching stable sputum culture at 12 weeks as this is often included in phase 3 trials as an early indicator of treatment response. A one-sided z-test was used to test the hypothesis that this proportion is smaller in the SOC arm ($\:{{\uppi\:}}_{1}$) arm compared to the proportion in the WGS arm ($\:{{\uppi\:}}_{2}$):

$$\:{\text{H}}_{0}:{\:{\uppi\:}}_{1}={{\uppi\:}}_{2},$$

$$\:{\text{H}}_{1}:{{\uppi\:}}_{1}<\:{{\uppi\:}}_{2}.$$

A significance level of 5% and a target power of 80% were considered to compare the sample sizes required by both methods.

### Imbalance imposed by randomization method

We simulated a randomization procedure for treatment allocation by random allocation and adaptive minimization using 1000 runs to calculate the imbalance in covariates in the study population. Potential important covariates for the efficacy of RR-TB treatment strategies included in the simulations were age (≤ 50 years, > 50years), semi-quantitive measure of mycobacterial load at baseline (four categories based on semi-quantitive Xpert Ct value or smear microscopy grading), history of second-line treatment for drug resistant TB (yes, no), body mass index (< 18 or ≥ 18), severity of disease (using hospitalization status at treatment initiation as a proxy), HIV status (six categories), contact with a known MDR-TB case (yes, no) and diagnosis of diabetes mellitus (yes, no). The four categories of semi-quantitive mycobacterial load were very low [Xpert Ct category very low, smear microscopy negative or scanty acid fast bacilli (AFB)], low (Xpert Ct category low or AFB 1+), medium (Xpert Ct Medium or AFB ++), or high (Xpert category high or AFB +++). The six HIV status categories were HIV status unknown, HIV negative, HIV positive not on antiretroviral treatment (ART), uncontrolled HIV on ART (latest CD4 count ≤ 100 cells/mm^3^ or viral load (VL) ≥ 1000 cells/ml), controlled HIV on ART (latest CD4 > 100 and VL undetectable), or HIV positive on ART with missing CD4 and VL information.

We calculated the expected imbalance in these covariates for two treatment allocation methods (random treatment allocation and adaptive minimization) using the sample sizes obtained when using either the commonly used endpoint or the alternative marker of treatment response [12–14, 19, 20]. For the minimization method, we applied the biased coin minimization, with equal treatment allocation, using a measure of imbalance by range [20].

To estimate the imbalance, we denote $\:n$_*ijk*_ as the number of patients already assigned to a treatment k (k = 1, 2, …, K), at levels j (j = 1, 2,…, J) of covariate i (i = 1, 2,., C). Assume that a new patient has levels r_1_, r_2_, …, r_C_ with regard to covariates 1, …, C, respectively, then denote the total number of participants at these levels for treatment k as $\:{n}_{{ir}_{i}\text{k}}$. The marginal imbalance on each level of these covariates is then measured by the absolute difference in number of patients in those groups between the two treatment arms:

$$\:\vert\:{\mathrm n}_{{\mathrm{ir}}_{\mathrm i}1}-{\mathrm n}_{{\mathrm{ir}}_{\mathrm i}2}\vert$$

The total imbalance $\:\text{G}$ is defined as the sum of the level-based imbalance, measuring the total imbalance between treatment 1 and 2 on each level r_1_, r_2_, …, r_C_ of the participant’s covariates 1, 2, …C:

$$\:\text{G}={\sum\:}_{\mathrm i=1}^{\mathrm C}{\mathrm w}_{\mathrm i}\vert\:{\mathrm n}_{{\mathrm{ir}}_{\mathrm i}1}-{\mathrm n}_{{\mathrm{ir}}_{\mathrm i}2}\vert$$

Weights can be pre-specified (i.e., w = (w_1_, w_2_, …, w_C_)) to indicate the relative importance of covariates in achieving balance. Because this is largely unknown for RR-TB we assumed equal weights for all covariates [20]. If this assumption were incorrect and some covariates were more critical for balance than others, underweighting the most influential covariates could lead to residual imbalance.

To quantify the expected imbalance, covariate values were sampled with replacement from data of the first 128 participants enrolled in the SMARTT-trial for a given sample size [15]. Participants with specific characteristics were then either randomly allocated or allocated using the *miniRand* package (minimization method) in R [20]. Imbalance was calculated for each sample and each allocation method. In total, 1000 simulation sets were created. As total imbalance is heavily dependent on the sample size, and thus not directly comparable between two samples; we calculated relative median imbalance for all scenarios; which is the total imbalance G divided by sample size n.

## Results

### Hypothesized effect size for the different endpoints as markers of treatment response

**Fig. 2:**
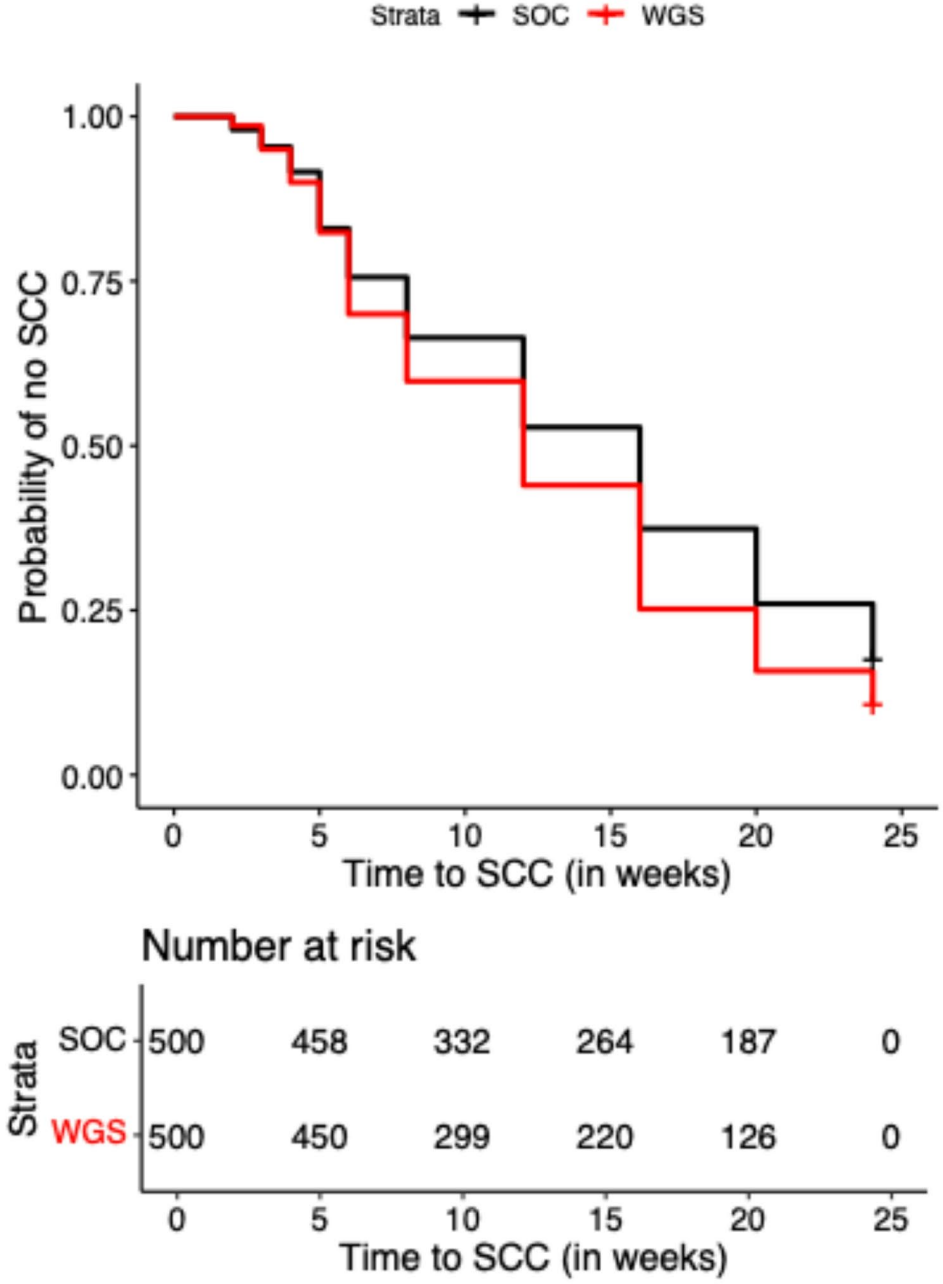
Kaplan-Meier curves for the time to stable culture conversion (SCC) (expressed in weeks) in the SOC (black solid line – standard of care) and WGS (red solid line - experimental) arms based on an effect size of a 28% reduction in MBL half-life in the WGS arm as compared to the SOC arm

To quantify the achieved power for the RCT with a given sample size, we first determined the effect sizes that represent a plausible difference between the two arms for the different endpoints as markers of treatment response. A 28% reduction in half-life of the MBL was considered a meaningful effect. This 28% reduction in MBL half-life corresponds to a 4-week reduction in median time to stable culture conversion (SCC), from 12 weeks in the SOC arm to 8 weeks in the WGS arm (Fig. 2). The 28% reduction in MBL half-life also corresponds to a 15% increase in the proportion of patients achieving SCC by week 12, from 60% in the SOC arm to 75% in the WGS arm. The 28% MBL half-life reduction also corresponds to a 12.8% increase in the proportion of participants achieving SCC by 6 months of treatment, from 83.2% in the SOC arm to 90.4% in the experimental WGS arm.

### Model configuration and simulated data

The parameter values obtained by calibrating the model to the characteristics of the expected study population (as per EXIT-RIF study) are shown in Table 1.

**Table 1:** Parameter values (and standard errors) for the semi-mechanistic model used in the base case scenario for the simulation approach. Parameter values are obtained after calibration based on data of the EXIT-RIF study (17)

Description	Parameter	Value (s.e.) reported in (6)	Value (s.e.) used in this study
MBL in patients			
Mycobacterial load (MBL) (n bacteria/inoculum)	$\:\text{M}\text{B}{\text{L}}_{0}^{\text{*}}$	2.14e3 (72.1)	933.58 (1.29)
Half-life MBL (in weeks)	mHL	0.81	2.03
Inter-individual variation in half-life MBL	$\:{{\upsigma\:}}_{\text{I}}^{2}$	0.33	0.33
Box-Cox transformation parameter	BXP	0.66	0.66
Standard of care (SOC) effect on mean half-life	$\:{{\upbeta\:}}_{\text{S}\text{O}\text{C}}$	−1	−0.50
Experimental (WGS) treatment effect on mean half-life	$\:{{\upbeta\:}}_{WGS}$	−1.42	−0.28
Baseline time to positivity (TTP) effect on$\:\text{M}\text{B}{\text{L}}_{0}^{\text{*}}$	$\:{{\upgamma\:}}^{\text{*}}$	–3.69 (0.03)	−4.13 (0.03)
Probability of bacterial presence			
Maximal probability of bacterial presence	$\:{\text{P}}_{\text{m}\text{a}\text{x}}$	0.97	0.97
MBL associated with 50% of$\:{\text{P}}_{\text{m}\text{a}\text{x}}$	$\:\text{M}\text{B}{\text{L}}_{50}$	0.50	0.50
Inter-occasion sputum sampling MBL variability	$\:{{\upsigma\:}}_{\text{S}}^{\text{*}2}$	2.89	2.89
Mycobacterial growth in MGIT system			
Growth rate (in 1/[day$\:\times\:$bacterial])	$\:{\text{k}}_{\text{G}}$	1.38e-6	1.38e-6
Carrying capacity in growth model ($\:\text{n}$bacteria)	$\:{\text{B}}_{\text{m}\text{a}\text{x}}^{\text{*}}$	4.76e5 (21215.52)	207733.7 (286.72)
Scaling of the hazard function	$\:{\text{h}}_{\text{s}\text{c}\text{a}\text{l}\text{e}}^{\text{*}}$	9.52e-5 (1.71e-6)	1.558e-6 (2.92e-9)

### Sample size calculation

**Fig. 3:**
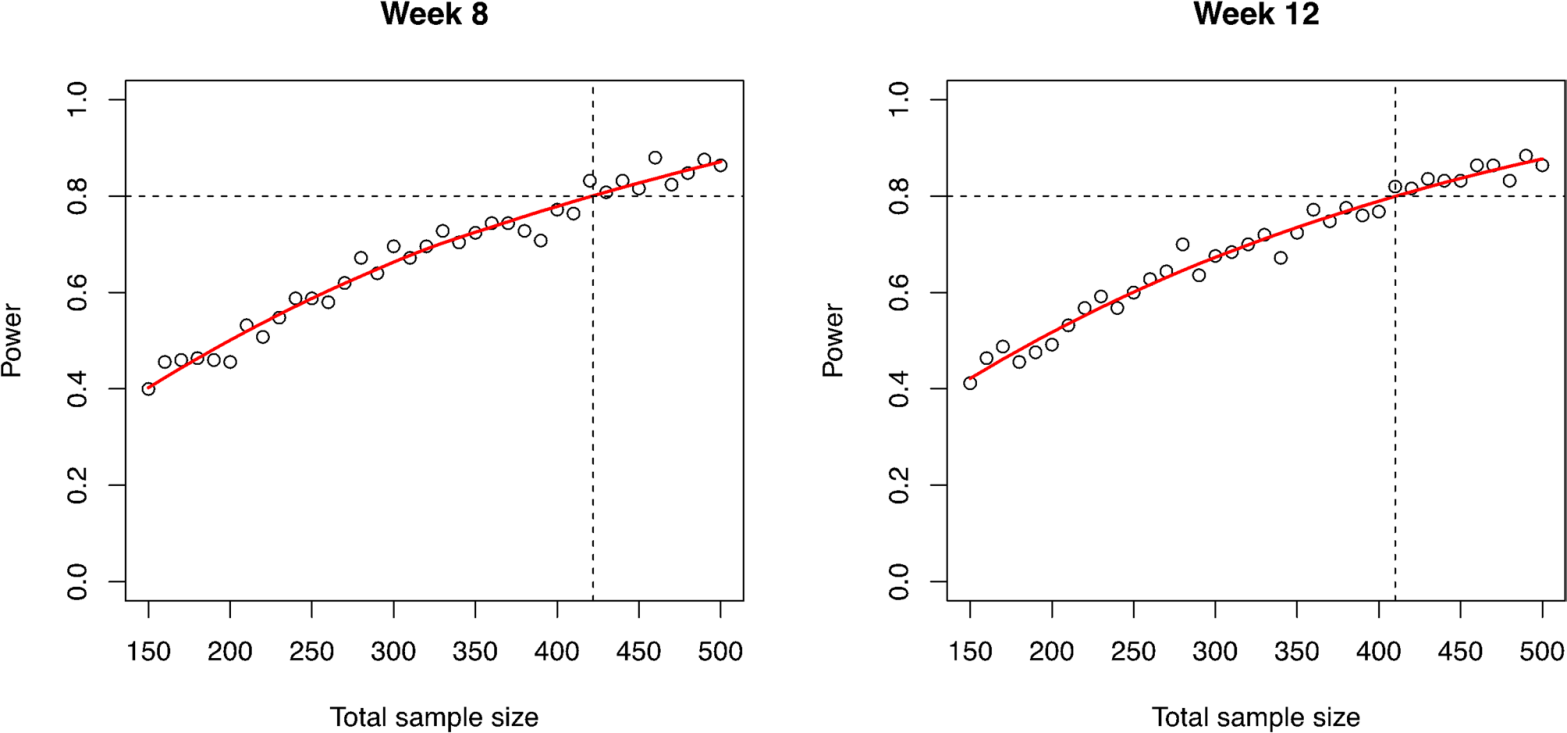
Observed (black dots) and smoothed (red solid line) relationships between the simulated total sample size (1:1 ratio between arms) and power to test the hypothesis (using a one-sided z-test) of a higher proportion of patients with stable sputum conversion at week 12 in the experimental (WGS) versus standard of care (SOC) arm

Using the model parameter values, their distributions, and the hypothesized effect size (Table 1), we first generated M = 250 samples for each given sample size to determine the power of the RCT when using the proportion of participants with SCC at 12 weeks. Simulations showed that to achieve 80% power to detect a difference of 15% in the proportion of participants reaching SCC (60% in the SOC-arm versus 75% in the experimental WGS-arm) a total sample size of 410 (i.e., 205 per group) patients with RR-TB would be required (Fig. 3).

**Fig. 4:**
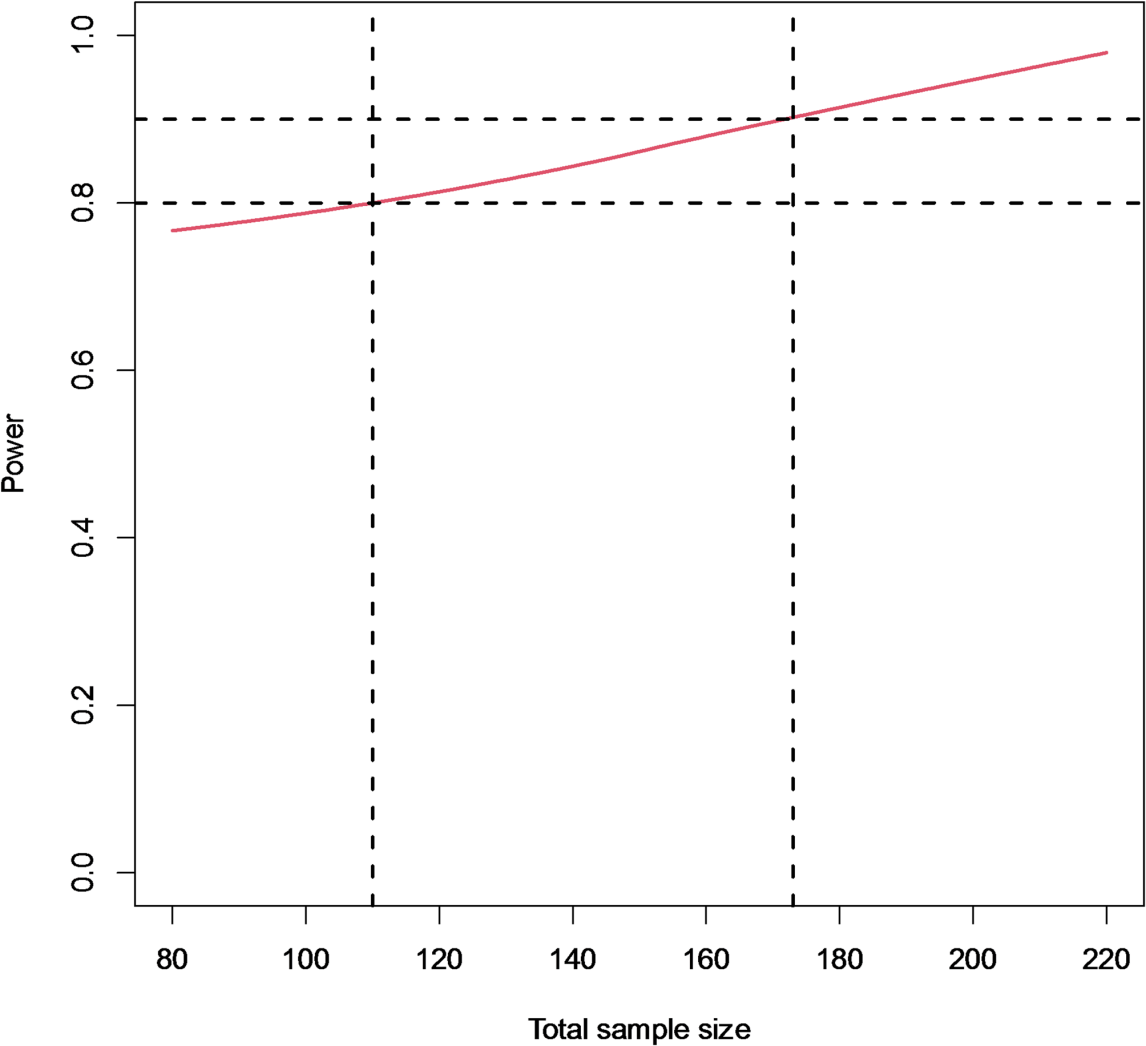
Smoothed (red solid line) relationship between the total sample size (equally balanced across both groups) and power of the (one-sided) Wald test to test the hypothesis of greater reduction in mycobacterial load in the experimental (WGS) versus standard or care (SOC) arm using the non-linear mixed effect model. The red shaded area provides pointwise 95% Wilson-score confidence intervals for the achieved power

Next, we determined the achieved power for varying sample sizes when relying on the semi-mechanistic non-linear mixed effects model to estimate the treatment effect on the time to culture positivity as the alternative marker of treatment response. Simulations showed that, to obtain a power of 80% to detect a 28% reduction in half-life of the MBL between the two arms (or a 4-week reduction in median time to stable culture conversion (SCC)); a total of 110 (i.e., 55 per group) patients (95% confidence interval [59, 143] based on pointwise 95% Wilson-score CIs for the simulation-based power) with RR-TB would be required (Fig. 4).

### Imbalance by randomization method

**Fig. 5:**
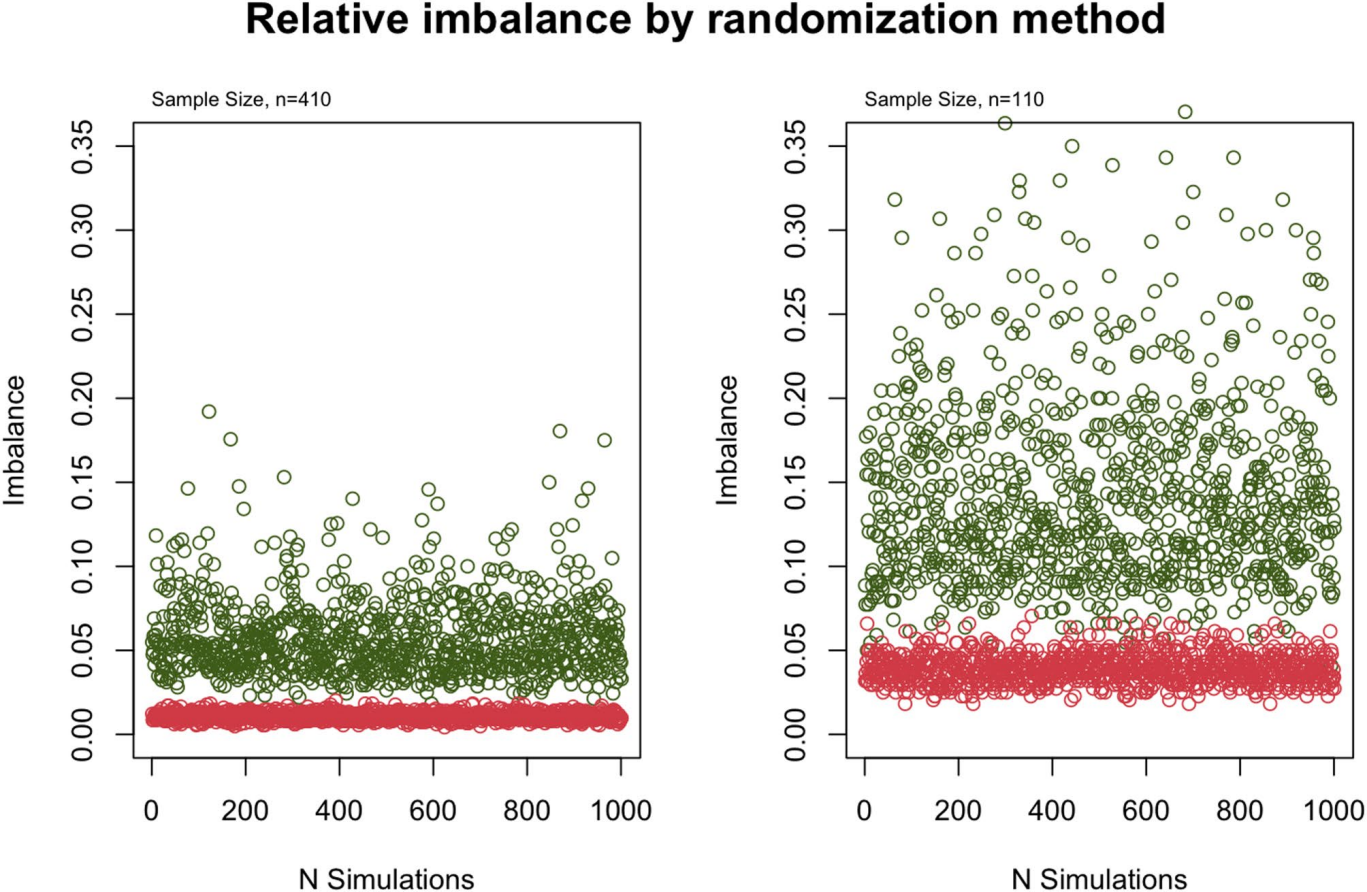
Relative imbalance obtained for 1000 simulated datasets with a sample size *n* = 410 (left panel) or *n* = 110 (right panel) and with 1:1 ratio of participants allocated to the experimental (WGS) or control (WGS) arm using either a random allocation procedure (green dots) or randomization via minimization (red dots)

When patients were randomly allocated to one of the two RCT arms, simulations showed a median expected imbalance of 11.50 [interquartile range, IQR 9.25–15.00.25.00] for a trial with a sample size of 110 and 21.75 [IQR 17.50–28.25.50.25] for a trial enrolling 410 participants. Relative to the sample size this relates to a median relative imbalance of 0.104 [IQR 0.084–0.143] for a sample size of 110 and 0.053 [IQR 0.044–0.071] for a sample size of 410 participants (Fig. 5).

When using adaptive minimization to randomize participants to one of the two treatment arms, simulations showed a median expected imbalance of 4.00 [IQR 3.5–4.5] for a sample size of 110 participants and 4.25 [IQR 3.75–5.25] for a sample size of 410 participants. Relative to the sample size this relates to a median relative imbalance of 0.036 [IQR 0.032–0.041] for a sample size of 110 and 0.010 [IQR 0.009–0.012] for a sample size of 410 participants.

## Discussion

RCTs are considered the gold standard for generating high-quality evidence. Unfortunately, RCTs often have large sample sizes making them complex, lengthy and costly endeavours, and placing them at risk of their results being less relevant in a rapidly changing policy environment [3, 4, 21–24]. In this study, we show that modelling the mycobacterial load over time using a semi-mechanistic non-linear mixed effects model, combining mathematical and statistical modeling aspects and relying on an alternative marker of treatment response derived from such a model, reduces the sample size of a clinical trial assessing the use of WGS on bacteriological response to RR-TB treatment by 73% (a total of 110 vs. 410 participants for a target power of 80%, one-sided hypothesis testing and specified significance level of 5%) as compared to a hypothetical trial using the proportion of participants reaching culture conversion by week 12 of treatment as an endpoint.

A smaller sample size is important to have timely results in rapidly evolving fields. In the first two decades after the first (1997) WHO guideline for treatment of drug resistant TB [25], new guidelines were published every two to six years [26–31]. Since 2018, guidelines have been updated yearly [32–35].

Trials should thus be as efficient as possible, which can be enhanced by using innovative trial designs and complex statistical approaches. Recently, adaptive trial designs are becoming more popular to improve efficiency [23–42]. One way to improve efficiency of RR-TB trials is by using a surrogate endpoint for relapse-free cure to overcome the need for 18 to 24 months of follow-up [43, 44]. Surrogate endpoints have been used in TB research for decades, but their predictive value remains uncertain [37, 43, 45, 46]. For example, in a treatment shortening trial using a fluoroquinolone-based regimen, the superiority based on surrogate endpoint analysis did not translate into improved clinical outcomes as patients in the experimentally treated group had higher rates of relapse [47, 48]. A recent study found that the week 8 culture conversion status performs poorly as a surrogate marker, whereas the week 24 performs better [45]. Based on work performed by Chigutsa et al. [7] and extended by Svensson et al. [6], we expanded their semi-mechanistic mixed-effects model to describe the change in mycobacterial load over time, to relate it to the time to culture positivity and turn this into an alternative indicator of treatment response. The increase in efficiency we observed can be explained by the use of all available individual-level culture data in a model compared to the commonly used endpoint of sustained culture conversion which is measured at a single time-point (i.e., week 12).

While more efficient, a smaller sample size can increase the risk of an unequal distribution of covariate factors between the two treatment arms [49, 50]. In our simulations, we indeed observed that the median relative imbalance increased from 0.053 for a sample size of 410 to 0.104 for a sample size of 110 individuals. We showed that this can be overcome by using an adaptive treatment allocation and randomization method [12–14, 19, 20], which reduced the median relative imbalance for a sample size of 110 from 0.104 to 0.036.

While ideally, sample size comparisons should be made to the clinical endpoints most commonly used in phase 3 trial, w could only simulate and compared change in MBL to another bacteriological endpoint. We opted for 12-week culture conversion as this is sometimes included in as a secondary endpoint in phase 3 trials. Demonstrating that MBL outperforms this established marker of bacterial response would supports its value as a potential surrogate for long-term outcomes in phase 3 settings and further research should evaluate the use of this novel surrogate endpoint [43, 51–54].

Another limitation is that fitting the semi-mechanistic mixed-effects model developed by Svensson et al. [6] to the simulated data requires advanced statistical knowledge. In the context of a trial and its sample size calculations, using MBL as an indicator of treatment response would involve re-calibrating the model to the expected trial population, simulating the population, and calculating the power for detecting treatment effects based on MBL and commonly used endpoints. While this adds complexity comes at the cost of an increased computational burden of the final trial analysis, this would be more than compensated for if the efficiency of the trial is increased.

We also note some technical limitations of the model. First, for simplicity reasons, a mono-exponential decay function was used for the evolution of the bacterial load. Hence, the proposed model may be vulnerable to misspecification of the parametric form used to describe MBL decay. If the true trajectory of bacterial clearance were to deviate substantially from the assumed mono-exponential model (for instance, more flexible functional forms such as a bi-exponential decline function, a Gompertz model [55] or a logistic model [56, 57] may better describe the growth of mycobacteria [7]) this could bias estimates of treatment effect and hamper interpretability of MBL-derived endpoints. In addition, the model relies on further parametric assumptions, including a log-logistic function to describe bacterial growth dynamics in the MGIT system, an acknowledged simplification of the complex in vitro proliferation process, and a semi-mechanistic linkage between bacterial burden and the hazard of culture conversion. While these assumptions enable efficient trial design and estimation, deviations from them could affect model predictions and reduce the robustness of conclusions, and future work should systematically verify all modeling assumptions.

Second, parameter values were derived from a combination of those determined in the original model developed by Svensson et al. [6], and re-estimation (calibration) of selected parameters to account for differences between study populations. This recalibration resulted in a longer estimated MBL half-life in our setting, which may reflect differences in disease severity, treatment history, and co-morbidities. Future research should formally assess the robustness of the observed relationship between MBL half-life and time to stable culture conversion, as well as establishing a range of plausible parameter values for all biological processes across different settings, to support the potential of MBL half-life as a valid surrogate endpoint.

Third, we note that in our simulations, the empirical type I error rate for a nominal α = 0.05 test was slightly inflated (7.6–8.8% across sample sizes). While this degree of inflation is modest, it underscores the importance of assessing and, where necessary, calibrating the analysis procedure, especially in settings where strict control of type I error is required. In such cases, when adaptive minimization is used for randomization, standard permutation-based inference is not directly applicable, and simulation-based calibration using the exact trial design and analysis procedure is recommended to empirically adjust decision thresholds or p-values.

Finally, we acknowledge that introducing endpoints as markers of treatment response, such as MBL, may face practical barriers to adoption in policy and clinical practice. While trust in MBL would grow alongside its methodological and clinical validation, a key challenge may lie in its divergence from routine monitoring strategies. Unlike time to culture conversion, which is a directly observeable endpoint that is independant from strong modeling assumptions [58], MBL is a model-derived quantity that may not yet be readily available or interpretable in programmatic settings [46]. This could hinder the uptake of trial results by policymakers and clinicians. Furthermore, the rapid pace of research and policy risks and the introduction of a new, less familiar indicator like MBL could result in losing their impact when implementation lags behind [59].

In conclusion, the use of change in mycobacterial load data over time as an alternative marker for bacteriological response to treatment can increase the efficiency of clinical trials evaluating new treatments for tuberculosis, especially when combined with an adaptive minimization strategy for randomization to overcome the increase in covariate imbalance associated with small trial sample sizes. While this is great news for notoriously difficult RCTs of RR-TB, proving the accuracy of this alternative marker for the prediction of the clinical endpoint of relapse-free cure will be important.

## Supplementary Information

Supplementary Material 1.

## Data Availability

The data and materials are available from the corresponding author on reasonable request.
