# Supplementary material for: Improving the efficiency of drug resistant tuberculosis treatment trials: a time-to-event alternative marker for bacteriological response and adaptive minimization for randomization
Source: BMC Med Res Methodol. 2025 Nov 25;25:265. doi: 10.1186/s12874-025-02697-w (PMC12649012; doi:10.1186/s12874-025-02697-w)
Supplement: Supplementary file 1 — Supplementary Material 1. [file 12874_2025_2697_MOESM1_ESM.docx]

Supplementary Material for the manuscript “Improving the efficiency of drug resistant tuberculosis treatment trials: a time-to-event alternative marker for bacteriological response and adaptive minimization for randomization”

Elise De Vos^1,2^, Annelies van Rie^1^, Steven Abrams^1,3^

1. Estimation of the model parameters in the mechanistic nonlinear mixed effects model

In order to estimate the model parameters of the mechanistic nonlinear mixed effects model, we rely on maximum likelihood estimation techniques. More specifically, based on (observed or simulated) patient-specific data $\boldsymbol{y}_{i}=(\boldsymbol{t}_{Gi}, \delta_{i},\boldsymbol{t}_{SSi}, \boldsymbol{t}_{STi})$, where $\boldsymbol{t}_{Gi}=(t_{Gi1},t_{Gi2},\ldots,t_{Gik_{i}})$ is the vector of observed times to positivity after inoculation in the MGIT system or the time of right censoring (in case the sputum sample turns out not to be positive after 42 days), $\delta_{i}=(\delta_{i1},\ldots,\delta_{ik_{i}})$ represents the vector of censoring indicator values (with value one in case of the true time to positivity being measured for a sputum sample, and zero otherwise) for observations 1 up to $k_{i}$ corresponding to individual $i=1, \ldots, n$. The vectors $\boldsymbol{t}_{SSi}$ and $\boldsymbol{t}_{STi}$ denote vectors of times since the start of the study and since treatment initiation, respectively, for individual $i$. Based on the observed (or simulated) data, the survival probability for individual $i$ (i.e., the probability of not having experienced positivity in the MGIT system at time $t_{G}$, conditional on time after the start of the study and treatment) is given by the expression:

$$S_{i}\left( t_{G} \right|t_{SSi},t_{STi})=\exp\left( -\int_{0}^{t_{G}} \lambda_{i}\left( t \right|t_{SSi},t_{STi})dt \right)=\exp\left( -h_{scale}\int_{0}^{t_{G}} B_{i}\left( t \right|t_{SSi},t_{STi})dt \right),$$

where $B_{i}\left( t \right|t_{SSi},t_{STi})$ refers to the number of bacteria present at the time after inoculation in the system.

For right-censored time-to-event data $\boldsymbol{y}_{i}$, the conditional loglikelihood contribution for patient $i$ (under the assumption of conditional independence of event times given the patient-specific random effects which are part of $B_{i}\left( t \right|t_{SSi},t_{STi})$) renders the form:

$${ll}_{i}\left( \boldsymbol{y}_{i} | \boldsymbol{\theta},\boldsymbol{b}_{i} \right)= \sum_{j=1}^{k_{i}} \delta_{ij}\left[ \lambda_{i}\left( t_{Gij} | t_{SSij},t_{STij},\boldsymbol{b}_{i} \right)S_{i}\left( t_{Gij} | t_{SSij},t_{STij}, \boldsymbol{b}_{i} \right) \right]+(1-\delta_{ij})\left( t_{Gij} | t_{SSij},t_{STij}, \boldsymbol{b}_{i} \right),$$

where $\boldsymbol{b}_{i}=\eta_{i}$ represents the vector of (latent) random effects associated with individual $i$ (here a single random intercept) and $\boldsymbol{\theta}$ is the vector of all model parameters. For ease of presentation, dependence on $\boldsymbol{\theta}$ is suppressed from the notation on the right-hand side of the equation.

A marginal (log)likelihood function for this parametric time-to-event model can now be formulated based on a marginalization of the conditional survival function (conditional on patient-specific random effects) as specified above, i.e.,

$$ll\left( \boldsymbol{y}_{i} | \theta\right)=\int_{-\infty}^{\infty} {ll}_{i}\left( \boldsymbol{y}_{i} | \boldsymbol{\theta},\eta_{i} \right)f_{r}\left( \eta_{i} \right)d\eta_{i},$$

with $f_{r}\left( . \right)$ the random effects distribution.

Marginalization is done in our case using numerical integration techniques. Following marginalization, the unknown model parameters are estimated using classical maximum likelihood estimation techniques applied to the marginal likelihood function, thereby providing maximum likelihood estimates for all model parameters, including the one quantifying the treatment effect.
